# Morphological anomalies of endangered Korean relict long-horned beetle *Callipogon relictus* (Cerambycidae, Coleoptera) during ontogenesis and possible causes of their occurrence

**DOI:** 10.3897/zookeys.714.21112

**Published:** 2017-11-06

**Authors:** Dae-Am Yi, Alexander V. Kuprin, Yeon Jae Bae

**Affiliations:** 1 Laboratory of Biodiversity and Ecology, Division of Environmental Science and Ecological Engineering, College of Life Sciences and Biotechnology, Korea University, 145 Anam-ro, Seongbuk-gu, Seoul, Republic of Korea; 2 Federal Scientific Center of the East Asia Terrestrial Biodiversity, Far Eastern Branch of the Russian Academy of Sciences, Vladivostok-22, 690022, Russia; 3 Laboratory of Biodiversity and Ecology, Division of Environmental Science and Ecological Engineering, College of Life Sciences and Biotechnology, Korea University, 145 Anam-ro, Seongbuk-gu, Seoul, Republic of Korea; 4 Center for the Study of Insect Ecology, Yeongwol Insect Museum, Donggang-ro 716, Yeongwol-gun, Gangwon-do, Republic of Korea

**Keywords:** *Callipogon relictus*, Korean relict long-horned beetle, morphological abnormalities, teratology

## Abstract

This paper describes for the first time cases of exogenous morphological anomalies that occur during rearing of *Callipogon relictus* Semenov, 1899 in a laboratory setting. The highest frequency of the anomalies has been observed during pupation. It can be assumed that in beetles of this group, at final stages of ontogenesis, some abiotic factors such as humidity and temperature play an important role.

## Introduction

Morphological anomalies are common in different groups of insects but there occurrence is scarce. A detailed classification and terminology of morphological anomalies and teratism in beetles was proposed by J. Balazuc (1948). Later, different authors described cases of atypical body structures (mostly in imago) in many groups of insects: lice (Blagoveshenskiy 1969), weevils (Stachowiak 1982, Chadwick and Brunet 1985, Cmoluch 1985, Read 1994, Nazarenko 2006, 2014), ground beetles (Ferreira 2008, Kamal et al., 2008), road beetles (Frank 1981, Ferreira 2011), leaf beetles (Prisniy 1983, Abdullah and Abdullah 1969), hymenoptera (Balazuc 1958, Akre et al. 1982), as well as specimens obtained during laboratory breeding (Savini and Furth 2004). Some authors noted morphological anomalies in some representatives of the family Cerambycidae (Balazuc 1952, Floch de Gallaix 1974, Cofais 1976, Osuna 1992, Schneider &Thoma 2004, Rahola 2005). For adults of *Stictosomus semicostatus* Audinet-Serville, 1832, *Ctenoscelis ater* (Olivier, 1795), *Enoplocerus armillatus* (L., 1767), and *Acanthinodera cumingii* (Hope, 1833), cases of antennal aberrations and deformations of the pronotum and elytra were described (Thouvenot 2006, Vitali 2007). In most cases, the causes of developmental anomalies of various parts of the body are still unclear but, following the outcomes of these experiments, some outwardly similar anomalies may be a consequence of various environmental factors (Vasilieva 2005).

The purpose of this paper is to describe the cases of morphological anomalies identified in the preimaginal stages and adult of *Callipogon relictus* that have been bred in a laboratory setting.

## Materials and methods

Specimens for this article were obtained during the course of elaboration of methods for laboratory breeding and maintenance of a rare representative of the family of long-horned beetles, *Callipogon relictus* Semenov, 1899, which is widespread in East Asia and is a rare and endangered species (Kim et al. 1976, Kuprin and Bezborodov 2012, Li et al. 2012, Kuprin 2016, Yi et al. 2017a). The elaborated methods of laboratory breeding of this species are detailed in our works (Kuprin et al. 2014, Yi et al. 2017b).

In addition, specimens collected in various habitats and species that are stored in the scientific institutions have been examined:

**ZIN RAS** Zoological Institute of the Russian Academy of Sciences, St. Petersburg, Russia,

**ZMMU** Zoological Museum of M.V. Lomonosov State University, Moscow, Russia,

**IBSS FEB RAS** Institute of Biology and Soil Science, Far East Branch of the Russian Academy of Sciences, Vladivostok, Russia,

**UNR FEB RAS** Ussuri Nature Reserve, Far East Branch of the Russian Academy of Sciences, Ussuriysk, Russia,

**KU** Korea University, Seoul, South Korea,

**YIM** Yangpyeong Insect Museum, Yangpyeong-gun, Gyeonggi-do, South Korea,

**PHS** Paichai High School, Seoul, South Korea,

**HRCI** Hampyeong Research Center of Insects, Hampyeong-gun, Junranam-do, South Korea,

**IZAS** Institute of Zoology, Chinese Academy of Sciences, Beijing, China.

The collection of adults and preimaginal stages obtained by us in the laboratory is stored in the Center for the Study of Insects Ecology of Yeongwol Insect Museum, Yeongwol, Korea.

## Results and discussion

**Figure 1.**
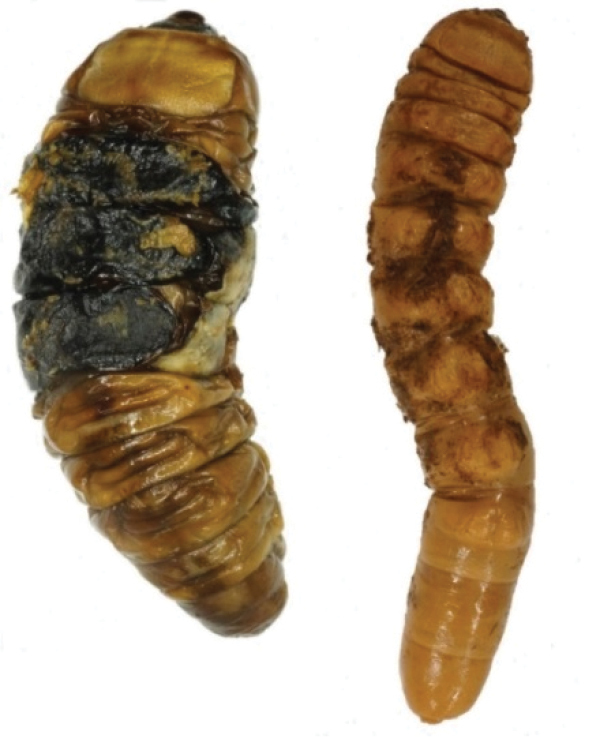
Larvae of *Callipogon relictus* with various deformations of the teguments.

**Figure 2.**
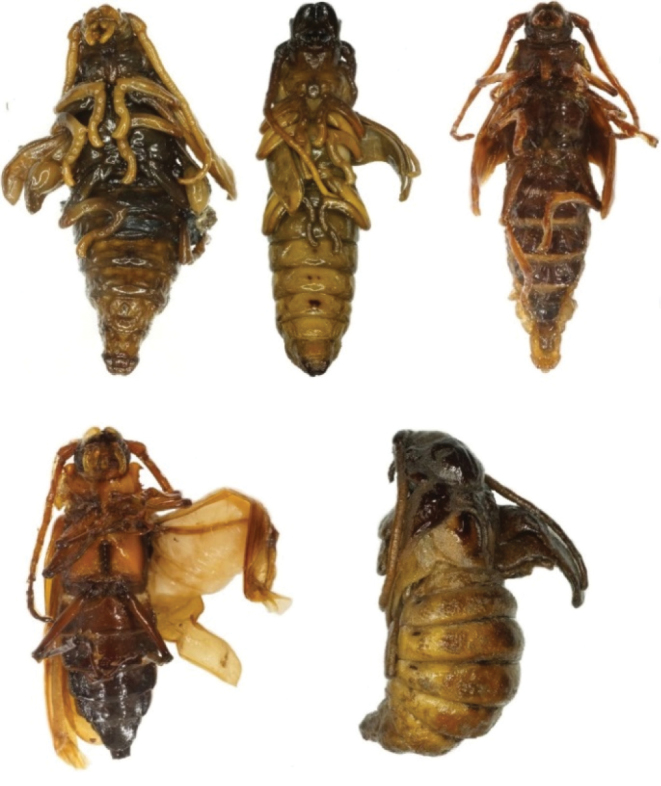
Pupae *C. relictus* with various deformations of teguments and limbs.

**Figure 3.**
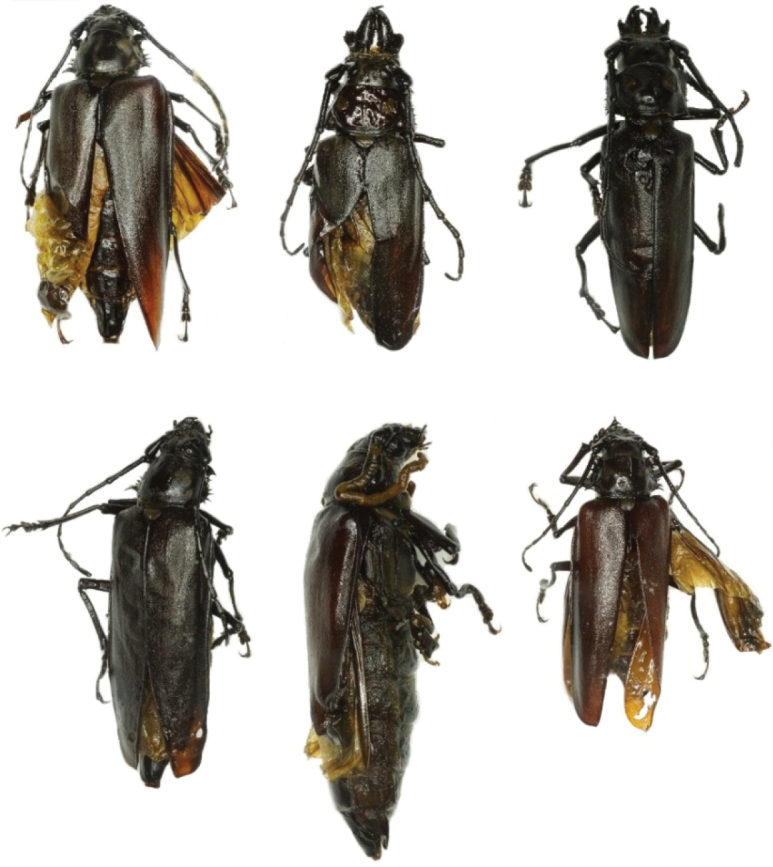
Adults of *C. relictus* with various developmental deformations of the body.

As a result of the investigation, more than 200 specimens of *Callipogon relictus* adults collected in various habitats and approximately 120 specimens of preimaginal stages and adults obtained in a laboratory setting have been studied. The most common anomalies in adults, larvae, and pupae have been grouped and are presented in Figures 1–3.

**Anomalies of larvae.** In the specimens studied, annular anomalies were identified of the body segments (Figure 1) and in some cases, isolated indurations in the form of tumours formed after molting of the larva and associated with delayed sclerotization of the teguments. It can also be assumed that occurrence of these anomalies is due to activity of xylobiontic mites. Notably, after a certain time, the larvae shown in Figure 1 started to lose weight and eventually died.

**Anomalies of pupae and adults.** Figures 2 and 3 show various complex anomalies of pupae and imago of *Callipogon relictus*. The most common anomaly in the adults (both in a laboratory setting and in nature) is the trematelytria, i.e., perforation of the elytra as a result of local necrosis of hypoderm before the formation of imaginal cuticle (Fig. 3). Similar cases are frequent in representatives of other groups of beetles, e.g., *Carabus cancellatus* Ill., *Silpha carinata* Hbst., *Silpha obscura* L., *Tenebrio molitor* L. (Prisniy 2009). There have been cases of brachelytria, i.e., shortening or reduction of the distal part, a decrease in the size of the elytra and, as a consequence, incomplete expansion of the elytra when the adult emerges. In a laboratory experiment, crossing of a male with brachelytria with a normal female led to development of larvae, which produced adult without morphological anomalies. In addition to the above-mentioned anomalies of the elytra, other deformities of the appendages of the body (mandibles, antennae, palps, and legs) have been found in adults (Fig. 3).

In the course of inspection of the collection material from the ZIN RAS, a dwarf specimen was found (1 male, Primorsky (Ussuriisky) krai, village of Yakovlevka, 23.VII.1926, D. Filipjev det.) with a body length of 3.18 cm. Dwarfism in insects can be caused both by exogenous factors (food deficiency and decrease in average temperatures and humidity during ontogenesis, population density) and endogenous factors (cumulative effect of many genes or pathologies of endocrine glands) (Wigglesworth 1967).

The nature of the described cases of morphological anomalies of *C. relictus*, as well as analysis of the literature data (Ortuño and Hernández 1993), suggest that they can occur in all representatives of the Prioninae. The most common deviations include all cases of deformation of elytra, perforation of elytra, disorder of innervation, and abnormalities of the legs (nearly 80% of the specimens studied). These investigations have shown that, as a rule, occurrence of such anomalies is caused by a change in the temperature and humidity regime or by mechanical damage to larvae and pupae (during biomorphological measurements).

It can be assumed that in natural conditions the occurrence of anomalies at the late stages of development of this species is also associated with a sharp change in the microclimate inside the pupal chamber built by instar-VI, or with a partial and complete destruction of the chamber by both animals and plants. In case of reduction in humidity, the pupal teguments dry up, which makes it difficult to release the adult from the exuvia residues and consequently, anomalies occur in total or partial deformation of the elytra, atrophy of the limbs, antennae and other appendages of the body. The increase in humidity, when the beetle has already been formed, leads to its death or damages to the integument in the process of sclerotization of the teguments in young beetles and to the development of various hematomas and indurations in the form of tumors and warts in larvae. It should also be noted that in a laboratory setting, pupae undergo a rhythmic change in color (the chestnut brown color of the body becomes lighter or darker) if the humidity level changes. Spraying of pupae with distilled water leads to darkening of the teguments within 40–50 minutes, and when the moisture level decreases, the color of the body becomes lighter. This phenomenon can explain the presence of dark specimens of imago in some collections (Korean peninsula) in contrast to northeast China and the south of the Russian Far East where chestnut brown samples have been found.

## References

[B1] Abdullah M Abdullah A (1969) Abnormal elytra, wings and other structures in a female *Trirhabda virgata* (Chrysomelidae) with a summary of similar teratological observations in the Coleoptera. Deutsche Entomologische Zeitschrift 16: 405–409. https://doi.org/10.1002/mmnd.19690160412

[B2] Akre RD Catts EP Zack RS Klostermeyer XC (1982) Gynandromorphs of *Megachile otundata* (Fab.) (Hymenoptera: Megachilidae). Entomological News 93(3): 85–94.

[B3] Balazuc J (1948) La tératologie des coléoptères et expériences de transplantation sur *Tenebrio molitor* L. Mémoires du Muséum National d’Histoire Naturelle 25: 1–293.

[B4] Balazuc J (1952) Un *Ergates faber* L. gynandromorphe (Col. Cerambycidae). Bulletin de la Société Entomologique de France (3): 34–38.

[B5] Balazuc J (1958) The tératologie des Hyménopteroides. Annales de la Société Entomologique de France 126: 167–203.

[B6] Blagoveshenskiy DI (1969) To the study of structural anomalies in lice (Siphunculata) Entomological Review 48(3): 507–510.

[B7] Chadwick CE Brunet BL (1985) Teratology in two species of beetles (Coleoptera). Victorian Naturalist 103(3): 106–108.

[B8] Cmoluch Z (1985) Weitere interessante teratologische Falle bei *Sitona lineatus* (L.) und *Dorytomus tremulae* (Payk.) (Curculionidae, Coleoptera). Polskie Pismo Entomologiczne 55(4): 819–823.

[B9] Cofais M (1976) Un cas de schistomélie ternaire chez un *Plocaederus* (Col. Cerambycidae). L’Entomologiste 32(6): 233–234.

[B10] Ferreira RN (2008) A teratological specimen of *Calosoma sycophanta* (L.), (Coleoptera; Carabidae) from Connecticut, USA. Entomological News 119(3): 307–309. https://doi.org/10.3157/0013-872X(2008)119[307:ATSOCS]2.0.CO;2

[B11] Ferreira RN (2011) Three anomalies of Coleoptera (Carabidae, Staphylinidae, and Scarabaeidae) from Connecticut. Insecta Mundi 0169: 1–3.

[B12] Floch de Gallaix P (1974) Cas de tératologie observé chez le *Cerambyx velutinus* (Col. Cerambycidae) de Provence. L’Entomologiste 30(1): 24–25.

[B13] Frank JH (1981) A revision of teratology in Staphylinidae with descriptions of a teratological specimen of *Tachinus axillaris* Erichson (Coleoptera, Staphylinidae: ) from Florida. Florida Entomologist 64(2): 337–340.

[B14] Kamal J Gandi K Hermes DH (2008) Report on the largest occurrence of morphological anomalies in ground beetles (Coleoptera, Carabidae). The Coleopterists Bulletin 62(1): 104–113. https://doi.org/10.1649/1032b.1

[B15] Kim CW Yoon IB Nam SH (1976) On the habitats and habits of *Callipogon relictus* S. (Col. Cerambycidae). Journal of Korean Association for Conservation of Nature 11: 5–16.

[B16] Kuprin AV (2016) The longicorn beetles (Insecta, Coleoptera: ) of the Ussuri Nature Reserve and adjacent territories. Far Eastern Entomologist 309: 21–28.

[B17] Kuprin AV Bezborodov VG (2012) Areal of *Callipogon relictus* Semenov, 1899 (Coleoptera, Cerambycidae) in the Russian Far East. Biology Bulletin 39(4): 387–391. https://doi.org/10.1134/S1062359012030090 22988764

[B18] Kuprin AV Bezborodov VG Yi DA Kotlyar AK (2014) Developmental biology and ecological peculiarities of the relict longhorn beetle *Callipogon relictus* Semenov, 1899 (Coleoptera, Cerambycidae). Entomological Review 94(9): 1251–1256. https://doi.org/10.1134/S0013873814090061

[B19] Li J Drumont A Xueping Z Meixiang G Wei Z (2012) The checklist of Northeast China´s subfamily Prioninae and biological observation of Callipogon (Eoxenus) relictus Semenov-Tian-Shanskij, 1899 (Coleoptera, Cerambycidae, Prioninae). Les Cahiers Magellanes 9: 50–56.

[B20] Nazarenko VY (2006) A Case of Teratology in Weevil Hypera transsylvanica (Coleoptera, Curculionidae). Vestnik zoologii 40(2): 181–183.

[B21] Nazarenko VY (2014) Morphological anomalies in Molytinae weevils (Coleoptera, Curculionidae). Ukrainian Entomological Journal 1(8): 69–72.

[B22] Ortuño VM Hernández JM (1993) Diversos casos teratológicos en Coleoptera. Boletín de la Real Sociedad Española de Historia Natural (Sección Biológica) 89(1–4): 163–179.

[B23] Osuna E (1992) Fenomeno teratologico en *Psalidognathus* sp. (Coleoptera: Cerambycidae). Boletín de Entomología Venezolana NS 7(2): 145–156.

[B24] Prisniy JA (2009) Classification of morphological abnormalities of beetles (Coleoptera). Belgorod State University Scientific Bulletin 9(11): 72–81.

[B25] Prisniy AB (1983) Morphological abnormalities Colorado potato beetle *Leptinotarsa decemlineata* Say (Coleoptera, Chrysomelidae). Entomological Review 52(4): 690–701.

[B26] Rahola P (2005) La collection Jean-Philippe Lamour (1935–2001) (2^e^ note). L’Entomologiste 61(6): 253–254.

[B27] Read RWJ (1994) An unusual specimen of *Sciaphilus asperatus* (Bonsdorff) (Curculionidae). The Coleopterist 3(1): 23–24.

[B28] Savini V Furth D (2004) Teratology in Coleoptera: a case in *Gioia bicolor* (Blake 1969) (Chrysomelidae, Alticinae) from Jamaica. Entomotropica 19(3): 165–167.

[B29] Schneider N Thoma J (2004) Malformation antennaire observée chez *Callichroma velutinum* (Fabricius, 1775) (Coleoptera, Cerambycidae). Bulletin de la Société des naturalistes luxembourgeois 105: 105–108.

[B30] Stachowiak P (1982) An interesting case of teratology in *Otiorhynchus rotundatus* Sieb. (Coleoptera, Curculionidae). Przegląd Zoologiczny 26(1): 115–117.

[B31] Thouvenot M (2006) Note sur trois anomalies antennaires chez des Prioninae Callipogonini de Guyane française (Coleoptera Cerambycidae). L’Entomologiste 62(1-2): 45–46.

[B32] Vasiljeva LA (2005) Changing wing venation of *Drosophila melanogaster* under the influence of temperature shock and selection. Biology Bulletin Review 66(1): 68–74.

[B33] Vitali F (2007) Anomalies multiples chez un exemplaire tératologique d’ *Acanthinodera cumingii* (Hope, 1833) (Coleoptera Cerambycidae). L’Entomologiste 63(2): 87–88.

[B34] Wigglesworth VB (1967) The Principles of Insect Physiology. 6th ed, Methuen and Co., London, 741 pp.

[B35] Yi DA Kuprin AV Bae YJ (2017a) First record of *Callipogon relictus* Semenov, 1899 (Coleoptera: Cerambycidae: Prioninae) from Lazovsky Nature Reserve, Primorsky region, Russia. Entomological news 126(5): 421–423. https://doi.org/10.3157/021.126.0512

[B36] Yi DA Kuprin AV Lee YH Bae YJ (2017b) Newly developed fungal diet for artificial rearing of the endangered long-horned beetle *Callipogon relictus* (Coleoptera: Cerambycidae). Entomological Research. https://doi.org/10.1111/1748-5967

